# Association of Central Serous Chorioretinopathy With Endogenous or Exogenous Corticosteroids: A Report of 2 Cases

**DOI:** 10.1210/jcemcr/luaf196

**Published:** 2025-09-12

**Authors:** Bing Pang, Qing Ni, Ya-jing Wang, Jian-ming Ba, Qi Ni, Kang Chen

**Affiliations:** Department of Endocrinology, The First Medical Center of the People's Liberation Army General Hospital, Beijing 100853, China; Department of Endocrinology, Guang’anmen Hospital of China Academy of Chinese Medical Sciences, Beijing 100054, China; Department of Endocrinology, Guang’anmen Hospital of China Academy of Chinese Medical Sciences, Beijing 100054, China; Department of Endocrinology, The First Medical Center of the People's Liberation Army General Hospital, Beijing 100853, China; Department of Endocrinology, The First Medical Center of the People's Liberation Army General Hospital, Beijing 100853, China; Department of Endocrinology, The First Medical Center of the People's Liberation Army General Hospital, Beijing 100853, China; Department of Endocrinology, The First Medical Center of the People's Liberation Army General Hospital, Beijing 100853, China

**Keywords:** central serous chorioretinopathy, Cushing syndrome, corticosteroids, cases

## Abstract

This study aimed to elucidate the potential relationship between central serous chorioretinopathy (CSC) and both endogenous hypercortisolism and the administration of exogenous corticosteroids. Case 1 involved a 39-year-old female patient who presented with blurred vision and metamorphopsia. Ophthalmologic examinations confirmed bilateral CSC. Biochemical and clinical evidence suggested hypercortisolism, and abdominal computed tomography revealed an adrenal adenoma, leading to a diagnosis of adrenocorticotropin-independent Cushing syndrome (CS). Postoperatively, a regression of serous retinal detachments was observed within 6 weeks. Case 2 referred to a 60-year-old male patient with hyperthyroidism and Graves orbitopathy who experienced vision loss after intravenous administration of 4.5 g of methylprednisolone over 10 weeks. Vision deteriorated after glucocorticoid therapy but improved 6 months later on discontinuation. Subsequently, the patient received peribulbar injections of triamcinolone acetonide, resulting in acute vision loss, with ophthalmologic examinations confirming CSC. After the cessation of exogenous corticosteroids, CSC resolved, and retinal pigment epithelium detachment also resolved at 3 months. Although causality cannot be definitively established with only 2 cases, the spontaneous resolution of subretinal fluid following corticosteroid withdrawal is highly indicative. The use of both endogenous hypercortisolism and exogenous corticosteroids is implicated as a risk factor for CSC, warranting increased vigilance from endocrinologists.

## Introduction

Central serous chorioretinopathy (CSC) is a retinal condition characterized by the seepage of fluid from the choroidal vasculature through a compromised retinal pigment epithelium (RPE), leading to the formation of a localized serous detachment of the neurosensory retina [1]. Patients commonly report symptoms such as blurred vision, metamorphopsia, micropsia, macropsia, and central scotoma. A population-based retrospective cohort study conducted in Asian populations estimated a mean age-adjusted incidence of 27 per 100 000 in men and 15 per 100 000 in women [2]. A cohort study reported that CSC affects 5.4 per 10 000 person-years for men and 1.6 per 10 000 person-years for women among corticosteroid users and nonusers, respectively, indicating that corticosteroid use may be a risk factor for CSC [3].

This report presents 2 CSC cases associated with endogenous hypercortisolism or exogenous corticosteroid exposure to contribute to the body of evidence implicating corticosteroids in the etiology of CSC.

## Case Presentation

### Case 1

**Figure 1.**
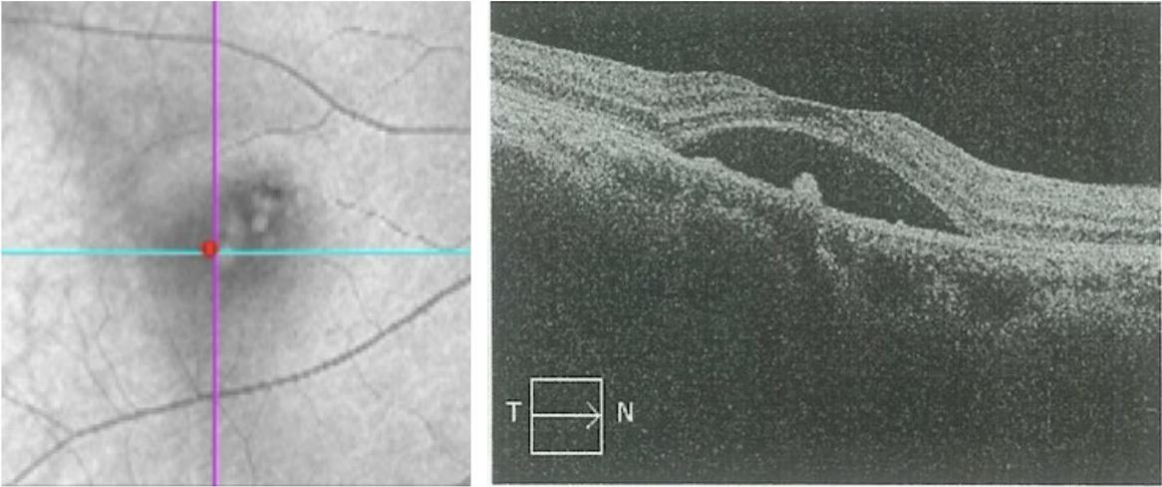
Optical coherence tomography of the right eye at presentation demonstrating macular edema and serous detachment of the neurosensory retina associated with retinal pigment epithelium detachment, with a subretinal fluid height of 436 μm, consistent with the diagnosis of central serous chorioretinopathy.

A 39-year-old female patient presented with a 7-month history of visual impairment in the right eye (RE) and 2 months’ progressive blurred vision and metamorphopsia. She exhibited progressive weight gain, round face, and hypokalemia. Computed tomography (CT) of the adrenal glands revealed an adenoma. Visual acuity at the time of presentation was 0.5 in the left eye (LE) and 0.6 in the RE, with a diagnosis of serous retinal detachment in the RE. Optical coherence tomography (OCT) of the RE demonstrated serous detachment of the neurosensory retina associated with RPE detachment (Fig. 1), consistent with CSC. The initial clinical signs suggested hypercortisolism, prompting referral to the endocrinology department for further examination.

### Case 2

**Figure 2.**
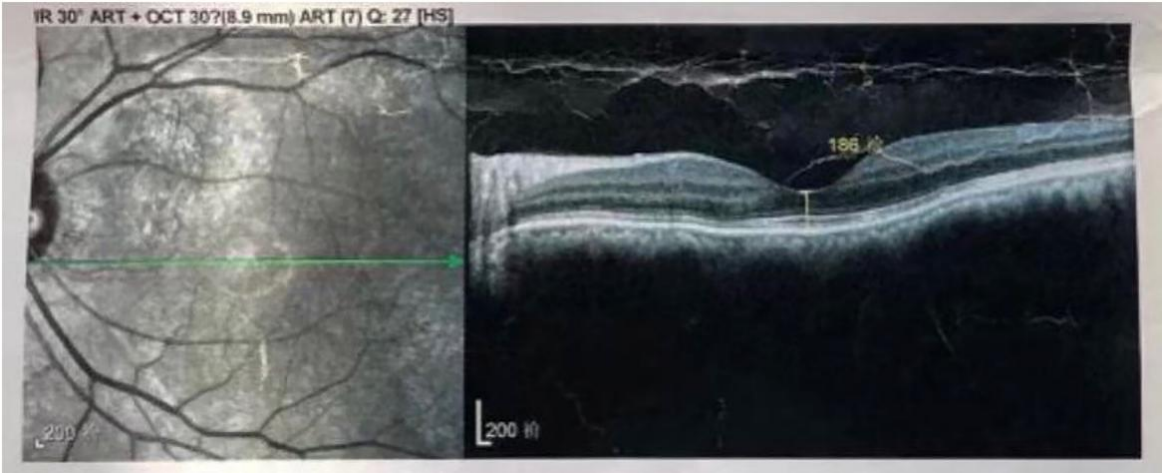
Optical coherence tomography (OCT) scan of the left eye of case 2. At the diagnosis of Graves orbitopathy, before triamcinolone acetonide injections. OCT images at presentation demonstrating a small amount of fluid accumulation beneath the neurosensory retina, with a subretinal fluid height of 186 μm.

A 60-year-old male patient with a history of hyperthyroidism and Graves orbitopathy (GO) presented with vision loss in the LE over the past 2 weeks. The patient was diagnosed with GO 1 year prior. At that time, OCT revealed a small accumulation of fluid beneath the neurosensory retina, leading to serous detachment (Fig. 2). He was admitted to the 900 Hospital of the Joint Service Support Force of the People's Liberation Army of China for intravenous glucocorticoid (GC) therapy, with a cumulative dose of 4.5 g of methylprednisolone, administered in 10 weekly infusions (3 consecutive days of infusions of 0.5 g during the first week, 3 weekly infusions of 0.5 g, followed by 6 weekly infusions of 0.25 g). Thyroid dysfunction was managed with 10 mg thiamazole given orally twice a day. The patient experienced blurred vision after intravenous GC therapy, which resolved after 6 months. Three months prior, the patient received 5 peribulbar injections of triamcinolone acetonide (TA) 40 mg every 2 weeks, leading to acute vision loss and metamorphopsia in the LE. The patient was referred to the endocrinology department for optimal treatment of GO.

## Diagnostic Assessment

### Case 1

**Figure 3.**
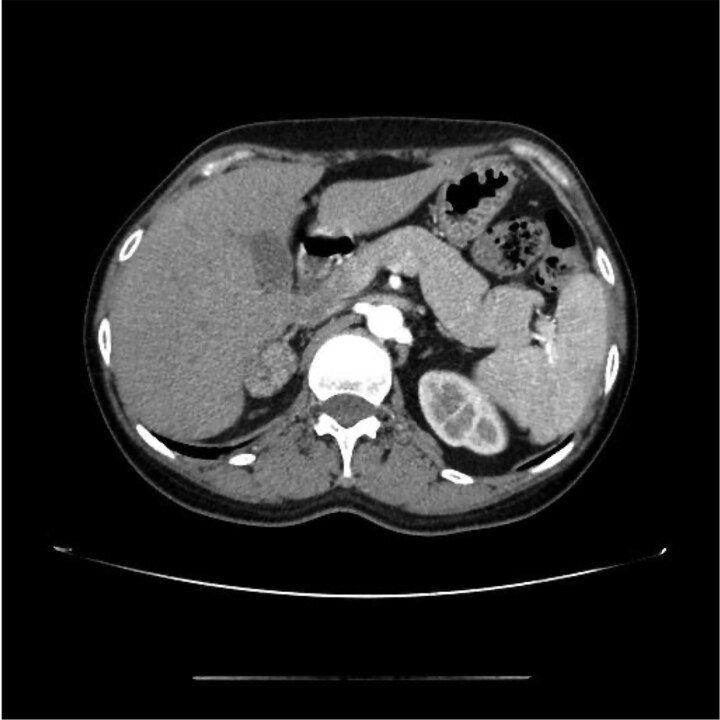
Abdominal computed tomography scan revealing a right adrenal oval-shaped adenoma measuring 29 × 21 mm with irregular enhancement.

The physical examination revealed hypertension (143/94 mm Hg), a weight gain of 5 kg over the past year (current weight, 55.6 kg), a moon-shaped face, increased vellus hair, subcutaneous ecchymosis, and thin skin on the limbs. First-line screening tests revealed a failure of morning cortisol suppression during a low-dose (1 mg) dexamethasone suppression test, with a cortisol level of 21.76 µg/dL (600.03 nmol/L) (normal reference range, < 1.81 µg/dL; < 50 nmol/L) and increased urinary free cortisol of 994.43 µg/24 hours (2744.62 nmol/24 hours) (normal reference range, 19.28-317.5 µg/24 hours; 53.2-876.3 nmol/24 hours). Details on the circadian rhythm of cortisol secretion and the low-dose dexamethasone suppression test are provided in Table 1. The patient was diagnosed with adrenocorticotropin hormone (ACTH)-independent CS. Plasma levels of aldosterone (11.6 ng/dL; 321.32 pmol/L; normal reference range in the lying position, 3.0-23.6 ng/dL; 83.1-653.72 pmol/L), renin (8.6 µIU/mL; 8.6 mU/L; normal reference range, 2.8-39.9 µIU/mL; 2.8-39.9 mU/L), and metanephrines (210.02 pg/mL; 106.5 pmol/L; normal reference range, ≤ 82.82 pg/mL; ≤420 pmol/L) and methoxyepinephrine (1.21 ng/mL; 380.6 pmol/L; normal reference range, ≤ 2.26 ng/mL; ≤710 pmol/L) were normal. Abdominal CT revealed a right adrenal oval-shaped adenoma measuring 29 × 21 mm with irregular enhancement (Fig. 3). Biochemical examination revealed hypokalemia (3.34 mEq/L; 3.34 mmol/L; normal reference range, 3.5-5.5 mEq/L; 3.5-5.5 mmol/L). The oral glucose tolerance test indicated increased postprandial blood glucose and insulin resistance (Table 2). Based on 24-hour blood pressure (BP) monitoring, the patient was diagnosed with grade 1 hypertension (average BP, 148/102 mm Hg), without prior antihypertensive drug use. The patient denied a history of hormone drug abuse and had not taken exogenous steroids.

**Table 1.** Circadian rhythm of cortisol secretion

	ACTH	F	UFC	Normal reference range
0 Am	<100.12 pg/mL (<2.2 pmol/L)	20.36 µg/dL (562.66 nmol/L)		ACTH (0.04 –0.31 pg/mL; 1.6-13.9 pmol/L)
8 Am	<100.12 pg/mL (<2.2 pmol/L)	23.37 µg/dL (646.04 nmol/L)	998.04 µg/24 h (2744.62 nmol/24 h)	F (4.81-19.43 µg/dL; 133-537 nmol/L)
4 Pm	147.45 pg/mL (3.24 pmol/L)	22.58 µg/dL (624.14 nmol/L)		UFC (19.3-318.7 µg/24 h/53.2-876.3 nmol/24 h)
LDDST	109.22 pg/mL (2.4 pmol/L)	22.15 µg/dL (612.35 nmol/L)	700.03 µg/24 h (1925.08 nmol/24 h)	

Abbreviations: ACTH: adrenocorticotropin hormone; F: cortisol; LDDST: low-dose dexamethasone suppression test; UFC: urinary free cortisol.

**Table 2.** Oral glucose tolerance test, 75 g

	Blood glucose	Insulin	C-peptide (nmol/L)	Normal reference range
0 min	75.06 mg/dL (4.17 mmol/L)	2.9 mU/L (17.40 pmol/L)	1.35 ng/mL (0.45 nmol/L)	Blood glucose (61.2-109.8 mg/dL; 3.4-6.1 mmol/L)
60 min	230.94 mg/dL (12.83 mmol/L)	141.8 mU/L (850.8 pmol/L)	13.77 ng/mL (4.59 nmol/L)	Insulin (2.6-24.9 mU/L; 15.6-149.4 pmol/L)
120 min	147.78 mg/dL (8.21 mmol/L)	111.4 mU/L (668.4 pmol/L)	15.99 ng/mL (5.33 nmol/L)	C-peptide (1.1-4.4 ng/mL; 0.37-1.47 nmol/L)

### Case 2

**Figure 4.**
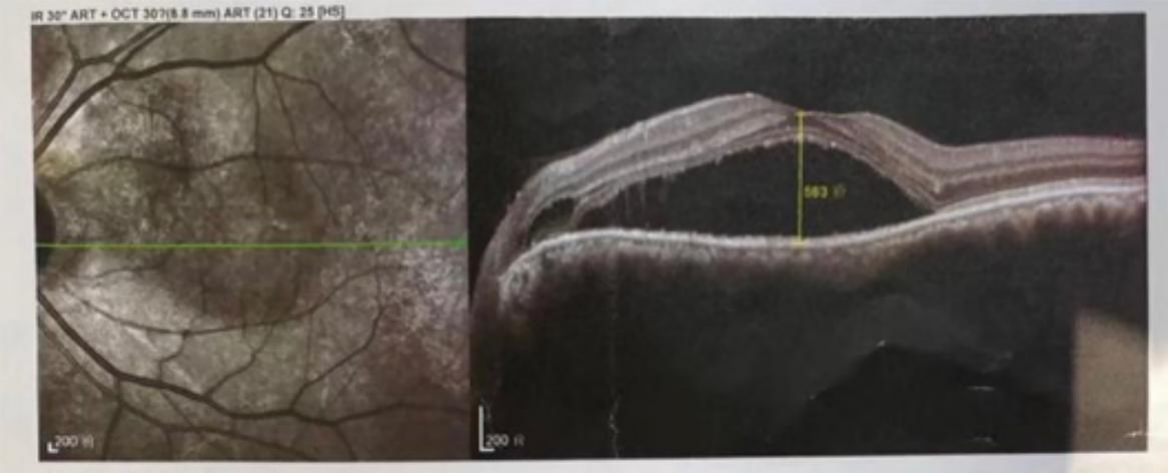
Optical coherence tomography (OCT) scan of the left eye of case 2. After triamcinolone acetonide injections. OCT images showing serous detachment of the neurosensory retina accompanied by a small retinal pigment epithelium detachment, consistent with the diagnosis of central serous chorioretinopathy, with a subretinal fluid height of 583 μm.

Visual acuities were 0.25 in the LE and 0.6 in the RE, with best-corrected visual acuity (BCVA) values of 0.3 in the LE and 1.0 in the RE. The intraocular pressure was 16.9 mm Hg in the LE and 18.0 in the RE. Physical examination revealed exophthalmos in both eyes, eyelid edema, conjunctival congestion, and diplopia in the right, upper-right, lower-right, and left gaze. OCT showed serous detachment of the neurosensory retina, accompanied by a small RPE detachment (Fig. 4). Thyroid function tests revealed a free triiodothyronine (FT3) level of 4.10 pg/mL (6.23 pmol/L; normal reference range, 1.82-4.14 pg/mL; 2.76-6.3 pmol/L), free thyroxine (FT4) of 11.98 pg/mL (15.57 pmol/L; normal reference range, 8.02-18.71 pg/mL; 10.42-24.32 pmol/L), and thyrotropin (TSH) of 0.01 µIU/mL (0.01 mIU/L; normal reference range, 0.35-5.5 µIU/mL; 0.35-5.5 mIU/L), with thyrotropin receptor antibody (TRAb) level of 13.05 U/L (normal reference range, ≤ 1.75 U/L). Orbital magnetic resonance imaging showed severe inflammation and hypertrophy in the superior, inferior, and internal rectus muscles, and thyroid ultrasonography suggested diffuse goiter and thyroid inflammation.

## Treatment

### Case 1

After evaluation and obtaining informed consent, the patient underwent right laparoscopic resection of the adrenal adenoma in the department of urinary surgery. Histological examination confirmed an adrenocortical adenoma.

### Case 2

The patient was advised to quit smoking and avoid light exposure. The methimazole dosage was adjusted to 10 mg once a day, and a selenium yeast tablet 100 mg once a day was added. Following evaluation, GC infusion was considered to have exacerbated CSC and RPE detachment; thus, we refrained from using this therapy. After providing informed consent, the patient was transferred to the department of ophthalmology for orbital decompression aimed at thyroid eye disease.

## Outcome and Follow-up

### Case 1

**Figure 5.**
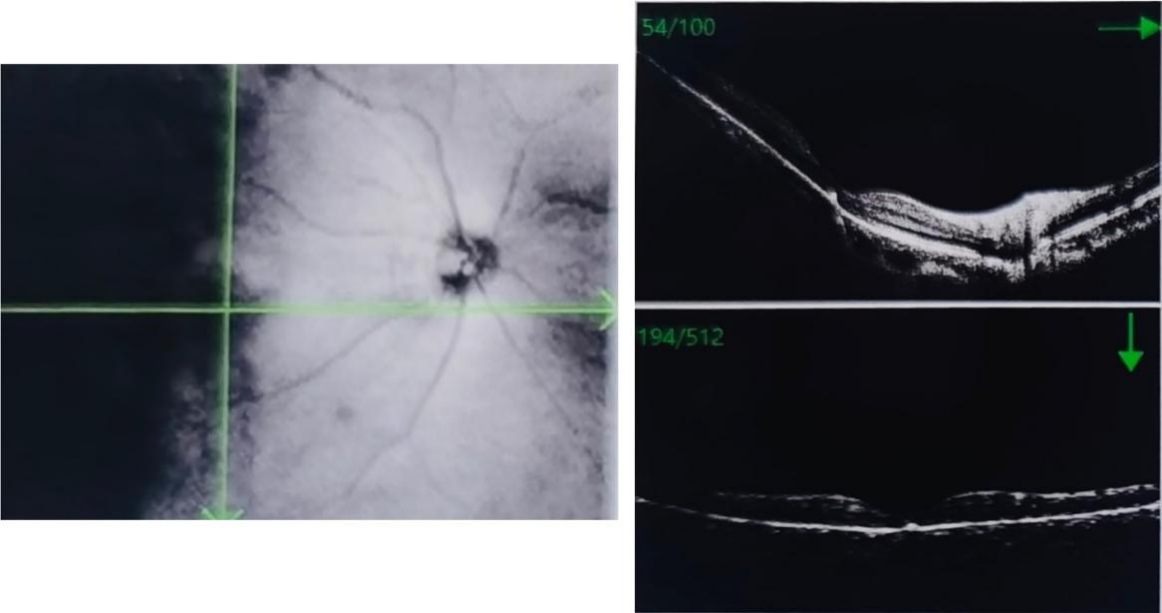
The retinal neuroepithelial layer has returned to normal, with a retinal thickness of 262 μm at the fovea of the macula.

Postoperatively, the patient received steroid replacement therapy. At 2 months postoperatively, the patient lost 3 kg, with regression of the moon-shaped face and subcutaneous ecchymosis. Laboratory data were as follows: cortisol, 0.06 µg/dL (1.66 nmol/L; normal reference range, 4.27-24.89 µg/dL; 118.02-687.96 nmol); ACTH, 0.91 pg/mL (0.02 pmol/L; normal reference range, 7.28-63.26 pg/mL; 0.16-1.39 pmol/L); fasting blood glucose, 89.82 mg/dL (4.99 mmol/L; normal reference range, 70.2-109.8 mg/dL; 3.9-6.1 mmol/L); K, 3.62 mEq/L (3.62 mmol/L; normal reference range, 3.5-5.5 mEq/L; 3.5-5.5 mmol/L). Visual acuities at the time of presentation were 0.8 in the LE and 0.7 in the RE. OCT showed regression of serous retinal detachments in the RE (Fig. 5).

### Case 2

**Figure 6.**
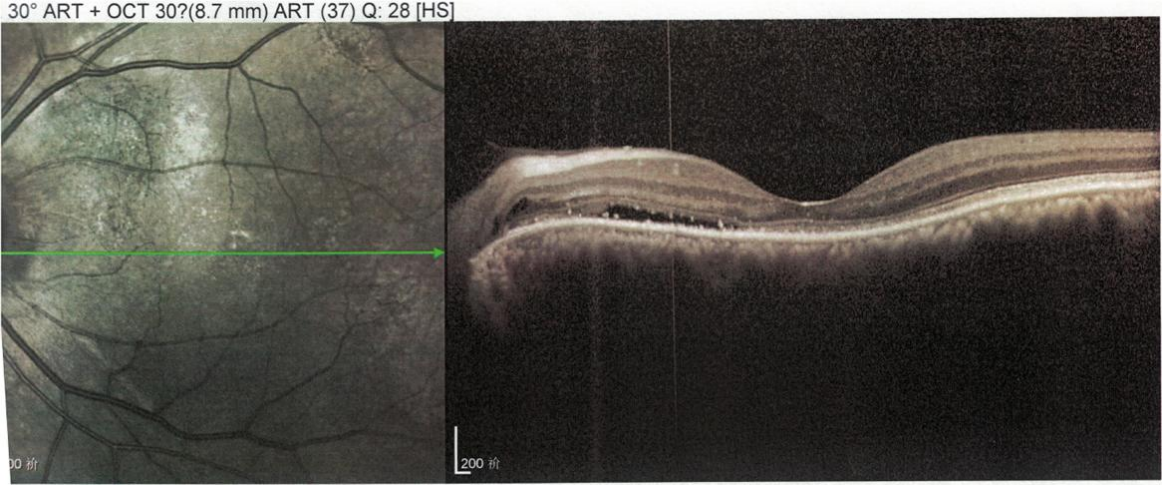
Discontinuing exogenous corticosteroid for 4 months, optical coherence tomography images at presentation showing improvement in subretinal fluid.

Three months after surgery, blurred vision symptoms improved. OCT revealed significant improvement in SRF compared with the preoperative status (Fig. 6). BCVA improved to 0.4 in the LE and 0.7 in the RE. In this case, drugs targeting CSC were not used; however, exogenous corticosteroids were discontinued. The patient monitored his thyroid function after discharge. Laboratory data revealed the following: FT3, 2.79 pg/mL (4.30 pmol/L; normal reference range, 2.27-2.69 pg/mL; 3.5-6.5 pmol/L); FT4, 7.97 pg/mL (10.36 pmol/L; normal reference range, 8.85-17.46 pg/mL; 11.5-22.7 pmol/L); and TSH, 2.558 µIU/mL (2.558 mIU/L) (normal reference range, 0.55-4.78 µIU/mL; 0.55-4.78 mIU/L), with TRAb, 11.81 U/L (normal reference range, ≤ 1.75 U/L), and methimazole dosage was reduced to 5 mg once a day.

## Discussion

CSC has been considered an initial presenting symptom of CS [4, 5]. To date, only 16 cases of CS combined with CSC have been reported [6-18]. In these cases (Table 3), the onset age ranged from 28 to 65 years, with a female predominance (12/16). Among them, 9 cases were caused by pituitary adenomas [7-10, 13, 16, 17], 4 by adrenal cortical adenomas [6, 11, 12, 14], and 1 each by adrenal nodular hyperplasia [7], adrenal cortical adenocarcinoma [15], and adrenal myeloid lipoma [18]. In terms of treatment, except for one case of death due to adrenal cortical adenocarcinoma, CSC symptoms and fundus features resolved after eliminating the causes (pituitary microadenoma resection or adrenal adenoma resection). A case of CSC associated with adrenal cortical adenomas, similar to case 1, has been described, with CSC as a presenting symptom of CS [11]. Bouzas et al [19] reported CSC development in 60 patients with CS, with 5% having 1 or more CSC episodes identified by ophthalmologic examination. CSC occurred during active CS with high plasma cortisol levels. Abalem et al [20] enrolled 11 patients with active CS and 12 healthy controls and found increased choroidal thickness in patients with active CS compared with controls (372.96 ± 73.14 vs 255.63 ± 50.70 µm), and 18.18% of patients experienced macular changes, possibly secondary to choroidal thickening. These results are generally consistent with those reported by Brinks and colleagues [21]. Therefore, ophthalmologists should consider CSC when patients with CS complain of blurred vision, and hormone levels must be evaluated to exclude an underlying endocrine disease.

**Table 3.** Literature review of Cushing syndrome associated with central serous chorioretinopathy

Author and reference	Age, y	Sex	Location of retinal lesions	Cortisol level	Location diagnosis	Prognosis
Soares et al [6]	50	Female	Right eye	Serum cortisol-, ACTH↓ and abnormal dexamethasone suppression test	Adrenal cortical adenomas	After undergoing minimally invasive adrenalectomy, SRF disappeared
van Dijk et al [7]	56	Male	Both eyes	Hypercortisolism	Pituitary adenomas	After undergoing transsphenoidal of pituitary microadenoma, no recurrence of serous SRF occurred
van Dijk et al [7]	49	Female	Both eyes	CS	Pituitary adenomas	After undergoing transsphenoidal adenectomy, visual symptoms and SRF did not recur
van Dijk et al [7]	37	Female	Left eye	ACTH-dependent CS	Pituitary adenomas	After undergoing transsphenoidal adenectomy, serous SRF was absent
van Dijk et al [7]	49	Female	Both eyes	ACTH-independent CS	Adrenal nodular hyperplasia	After undergoing bilateral adrenalectomy, no serous SRF was present
Buelens and Dewachter [8]	65	Female	Left eye	Serum ACTH-, 24-h urinary free cortisol↑, morning serum cortisol↑, failure to suppress morning cortisol secretion after overnight low dose of dexamethasone	Pituitary adenomas	After undergoing transsphenoidal tumor resection, visual acuity recovered and CSC regressed
Giovansili et al [9]	53	Female	Both eyes	Serum ACTH-, 24-h urinary free cortisol↑, morning cortisol was nonsuppressed with low-dose dexamethasone (1-mg DST)	Pituitary adenomas	Endoscopic endonasal transsphenoidal surgery was performed, visual acuity was recovered, and no retinal serous detachments existed
Wang and Saha [10]	49	Female	Both eyes	ACTH↑, nonsuppressed morning cortisol with 1-mg dexamethasone, 24-h urinary free cortisol↑	Pituitary adenomas	After undergoing transsphenoidal adenectomy, visual acuity recovered and CSC regressed
Iannetti et al [11]	45	Male	Left eye	ACTH↓, 24-h urinary free cortisol↑, morning cortisol was nonsuppressed with low-dose dexamethasone (1-mg DST) and high-dose dexamethasone (8-mg DST)	Adrenal cortical adenomas	After undergoing right laparoscopic adrenalectomy, SRF disappeared and visual acuity improved
Pastor-Idoate et al [12]	28	Male	Both eyes	ACTH↓, 24-h urinary free cortisol↑, morning cortisol was nonsuppressed with low-dose dexamethasone (1-mg DST) and high-dose dexamethasone (8-mg DST)	Adrenal cortical adenomas	After undergoing left laparoscopic adrenalectomy, visual acuity improved and RPE resolved
Clarke et al [13]	64	Male	Left eye	Morning serum cortisol↑	Pituitary adenomas	Patient received postoperative stereotactic radiosurgery to residual tumor, vision recovered, intraretinal edema and SRF resolved
Takkar et al [14]	42	Female	Both eyes	ACTH↓, no change in serum cortisol levels following low-dose dexamethasone suppression test	Adrenal cortical adenomas	After undergoing laparoscopic adrenalectomy, CSC resolved
Thoelen et al [15]	54	Female	Both eyes	ACTH↓, serum cortisol↑, urinary free cortisol↑, 17-hydroxysteroids and 17-ketosteroids ↑	Adrenal cortical adenocarcinoma	Despite total adrenalectomy, patient died of sepsis
Appa [16]	42	Female	Both eyes	24-h urinary free cortisol↑, low-dose and high-dose dexamethasone suppression test both failed to suppress serum cortisol level	Pituitary adenomas	Transsphenoidal endoscopic excision of pituitary microadenoma, recovery of vision, and CSC has not been explained
Liu Y et al [17]	58	Female	Both eyes	ACTH↑, plasma cortisol level↑	Pituitary adenomas	After undergoing transsphenoidal adenectomy, visual acuity recovered
Liu L et al [18]	44	Female	Both eyes	ACTH↓, plasma cortisol level↑, urinary 17-hydroxycorticosteroids failed to be suppressed by 8-mg dexamethasone	Adrenal myeloid lipoma	Left adrenalectomy was performed and visual acuity improved

Abbreviations: -, normal range; ACTH, adrenocorticotropin hormone; CS, Cushing syndrome; CSC, central serous chorioretinopathy; DST, dexamethasone suppression test; RPE, retinal pigment epithelium; SRF, subretinal fluid.

The use of exogenous corticosteroids is similarly the most recognized risk factor for CSC. Through a meta-analysis, Ge et al [22] found that administration of several forms of exogenous corticosteroids appeared to be associated with an increased risk (odds ratio [OR] 4.050; 95% CI, 2.270-7.220). In case 2, CSC initially developed after intravenous GC therapy, which improved after discontinuing corticosteroids. However, CSC was aggravated after receiving peribulbar injections of TA. Imasawa and colleagues [23] reported a similar case of presumed intravitreal TA-associated CSC in a 59-year-old female patient who had been treated with TA particles during vitrectomy to reduce macular edema. The use of corticosteroids was temporally associated with acute CSC, with patients experiencing complete resolution of CSC during a follow-up period of 2 months. The case published by Kocabora et al [24] involved a 42-year-old male patient who had been treated with intravitreal TA injection. Before exacerbating CSC, he had used TA 4 mg/0.1 mL for 3 weeks, and he experienced CSC resolution during a follow-up period of 6 months. A thick choroid is considered a risk factor for CSC development. Araki et al [25] reported that the choroidal thickness in patients with steroid-induced CSC was thicker than that in patients with idiopathic CSC; steroids can cause CSC through an effect on choroidal vessels and an impairment of RPE. The variant of complement factor H rs800292 A allele was associated with subfoveal choroidal thickness in normal participants [26]. Yoneyama et al [27] found a higher female predominance among steroid users than among nonsteroid users. Schellevis et al [28] observed high levels of androsterone, estrone, etiocholanolone, and androstenedione in patients with CSC compared with controls.

The etiological and pathophysiological link between corticosteroids and CSC has not been clear enough; however, possible mechanisms have been proposed. CSC development is found to be related to the abnormalities in choroidal circulation and alterations in the permeability of choroidal blood vessels. Corticosteroids have been hypothesized to inhibit collagen synthesis and increase choriocapillaris fragility and permeability, resulting in the decompensation of the choroidal circulation and accumulation of SRF. Corticosteroids are also thought to cause local ischemia by reducing choroidal fibrinolysis and leading to active pigment epithelial leakages or pigment epithelial detachment, resulting in SRF accumulation [22].

Although we cannot prove causality between CSC development and the use of endogenous hypercortisolism or exogenous corticosteroids, the spontaneous resolution of SRF following the discontinuation of corticosteroid therapy is very suggestive. Physicians should be cautioned that patients with CS or those who received high-dose steroid therapy are at risk of CSC.

## Learning Points

- CSC can be the main manifestation of unrecognized CS.
- Ophthalmologists should maintain a high level of suspicion for clinical signs of CS in patients with CSC.
- Exogenous corticosteroid usage is the most recognized risk factor for CSC.
- The spontaneous resolution of SRF accumulation following corticosteroid withdrawal is highly indicative.
- Patients with CS or those who received high-dose steroid therapy are at risk of CSC.

## Contributors

All authors made individual contributions to authorship. K.C. and Qi Ni were involved in the diagnosis and management of the patient and manuscript submission and manuscript conceptualization. B.P. and Qing Ni were involved in preparation of the case report and literature review and manuscript writing and submission. Y.J.W. was involved in reviewing the patient chart, collecting data, and preparation of histology images. J.M.B. was involved in manuscript reviewing, revision, and editing. All authors reviewed and approved the final draft.

## Data Availability

Data sharing is not applicable to this article as no data sets were generated or analyzed during the current study.

## Abbreviations

ACTH: adrenocorticotropin hormone
BCVA: best-corrected visual acuity
BP: blood pressure
CS: Cushing syndrome
CSC: central serous chorioretinopathy
CT: computed tomography
DST: dexamethasone suppression test
FT3: free triiodothyronine
FT4: free thyroxine
GC: glucocorticoid
GO: Graves orbitopathy
LE: left eye
OCT: optical coherence tomography
RE: right eye
RPE: retinal pigment epithelium
SRF: subretinal fluid
TA: triamcinolone acetonide
TRAb: thyrotropin receptor antibody
TSH: thyrotropin
